# Diagnosis and Management of Lentigo Maligna: Clinical Presentation and Comprehensive Review

**DOI:** 10.1155/2021/7178305

**Published:** 2021-07-24

**Authors:** Piyu Parth Naik

**Affiliations:** Department of Dermatology, Saudi German Hospitals and Clinics, Hessa Street 331 West, Al Barsha 3, Exit 36 Sheikh Zayed Road, Opposite of American School, Dubai, UAE

## Abstract

Lentigo maligna (LM), also known as Hutchinson's melanotic freckle, is a form of in situ melanoma characterized by the proliferation of atypical melanocytes along the basal epidermis in sun-damaged skin. If left untreated, LM will progress to lentigo maligna melanoma (LMM), a form of invasive melanoma with the same prognosis as other forms of invasive melanoma. LM is more common in the elderly, with a peak occurrence between the ages of 65 and 80 years. LM, however, is rarely present on the trunk and extremities. The diagnosis of LM, confirmed by histopathological and biopsy examination, is based on clinical and dermoscopic features. It typically begins as a tan-brown macule or patch, but it can progress to a variegated pigmentation with dark black color or even amelanotic characteristics. The risk factors involved in the LM development include a history of sunburns, lighter skin types, advanced age, history of nonmelanoma skin cancers, and tendency to form solar lentigines. This article explains the clinical presentation of LM, also reviews the available information on the diagnosis and management of LM, and discusses the potential of such information in facilitating the future prospective.

## 1. Introduction

Lentigo maligna (LM) is the noninvasive counterpart to lentigo maligna melanoma (LMM), which was first described by Hutchinson in the year 1890 [1]. LM appears on sun-damaged skin that has been exposed to the sun for a long time, most often on the head and neck. However, LM can be observed on the trunk and extremities on rare occasions [2]. Among melanomas, LM is unusual in that its natural history is that of an indolent, slow-growing tumor that can be present for years before being diagnosed. Even long-standing lesions are rarely invasive, and among all melanoma subtypes, LM has one of the highest 5-year survival rates, with an estimated 97.2 percent survival rate [3]. However, once invasive, LMM may be aggressive, increasing the risk of metastasis [4]. Overall, the subtype of LM accounts for around 10–26% of neck and head melanomas and 5–10% of melanomas, responsible for a large proportion of melanomas in patients above the age of 65 years [1]. However, according to Kasprzak and Xu [5], up to 30–50% of cases will progress to LMM if left untreated, with latency periods varying between 10 and 50 years. Although the LM to LMM latency is commonly thought to be over a decade, cases of LM to LMM progression in as little as 24 months have been recorded [6].

LMM is responsible for 5–15% of all cutaneous melanomas [1]. As the prevalence of this frequently difficult melanocytic neoplasm has recently risen, there have been debates on how to diagnose it and how to treat it. Between 1990 and 2000 in the United States, one study found a 52 percent rise in the incidence rate of LM among men and women aged 45 to 64 years [7]. Another research found that between 1970 and 1989, the incidence of cancer in the United States increased from 2.2 per 100,000 per year to 13.7 per 100,000 per year between 2004 and 2007 [8]. History of light skin, sun exposure, and a proclivity for lentigines are all risk factors for the development of LM. Unlike melanoma that spreads superficially, LMM is more closely linked to history of skin cancer and prior lentigines and is not linked to preexisting nevi or the likelihood of developing nevi [8]. Fair-skinned people with signs of actinic skin damage (actinic keratoses and solar lentigines) and nonmelanoma skin cancer have been linked to a higher risk of LM and LMM [8, 9]. In contrast to other subtypes of melanoma, ultraviolet radiation appears to play a vital role in its pathogenesis, with chronic rather than frequent sun exposure increasing the risk of LM [7]. The case report and current review aim to investigate the previous data on the diagnosis and management of LM/LMM.

## 2. Clinical Presentation and Dermoscopic Findings

LM/LMM typically appears as a large, pigmented macule (<10 mm) or patch (>10 mm) that grows slowly and has irregular borders. LM is almost always found on actinically damaged skin in clinical practice. Initially, it appears to be a small patch. Black, pink, darker brown, and light brown/tan are some of the colors that can be seen. Irregular borders, asymmetry, and a report of increasing size are all useful features for diagnosis.

**Figure 1:**
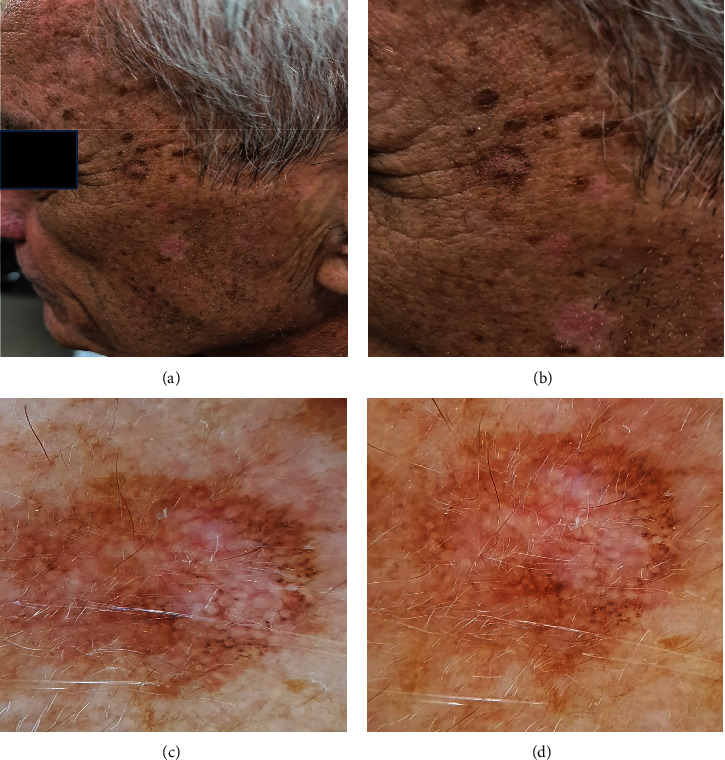
(a, b) Clinical presentation study showed an irregular pigmented flat macule on the left temporal area in the background of ageing skin. (c, d) Dermoscopy showed moth-eaten borders with a faint pigment network and circles within circles. There are irregularly distributed dots from the 3 o'clock to 6 o'clock position. Regression structures in the central area were noted.

The early sign of LM has been described as repigmentation of previously white or gray hair, and it may raise suspicion that LM in the scalp is a possibility [10]. Stolz et al. [11] were the first to characterize dermoscopic patterns unique to facial LMM. They developed an LM “progression model,” which identifies four stages of LMM invasion of hair follicles as seen by dermoscopy. Initially, hyperpigmented follicular openings occur (often irregularly). These characteristics relate histopathologically to the first visible signs of pigmented tumoral melanocyte invasion of the hair shaft. The annular-granular pattern is then created by fine globules and gray dots appearing around the follicles. Then, in the areas surrounding the hair follicle openings, rhomboid (lozenge-shaped) pigmented areas appear. Finally, as the hyperpigmentation coalesces, both follicular anatomical structures are infiltrated by malignant cells and the opening of follicle is obliterated. For the combination of these four characteristics, specificity and sensitivity were 93% and 89%, respectively [11, 12] (Figure 1).

## 3. Histopathology Analysis by Biopsy Technique

Histopathology analysis is typically used to render the diagnosis of LM/LMM. Common histological findings of lentigo maligna include confluent proliferation of melanocytes at junctions and their extension along adnexal structures. Associated solar elastosis is typically noteworthy [13]. Some researchers believe that the existence of melanophages can help distinguish LM from chronically sun-damaged skin melanocytic hyperplasia [14]. LM/LMM has atrophic epidermis, and basal keratinocytes may be hyperpigmented [15, 16]. There are no histological variations between extrafacial and facial LM/LMM, which is important to note [17]. In addition, spindle cell morphology is common during the vertical growth phase. Desmoplastic melanomas account for up to two-thirds of all desmoplastic melanomas, and neurotropism is widespread in highly invasive LMM. MART-1 and other immunohistochemical markers can aid in the detection of a dermal invasive component [14, 18].

When a lesion develops an invasive dermal component, nodularity becomes apparent. Seborrheic keratosis, pigmented basal cell carcinoma, pigmented actinic keratosis, lichen planus-like keratosis, and solar lentigo are among the clinical differential diagnoses [19]. The gold standard for diagnosing LMM is a histologic examination. Besides, to examine the entirety of the lesion, the complete excisional biopsy is most ideal and this technique is often ruled out due to prognostic factors such as a number of LMs with a large clinical size, maximum Breslow depth, and the most common position on the head and neck [1].

Melan A/MART1, SOX10, MITF, HMB45, and S100 are all melanocytic markers that have been used in the diagnosis of LM, particularly in cases where the diagnosis is unclear. Melan A is more precise, but it is not always effective in staining desmoplastic melanomas. S100 is the most sensitive stain, but it is also the least precise, which restricts its use [20, 21]. For the presence of melanocytic nuclear density greater than or equal to 9 *μ*m, MITF, a nuclear stain, has been shown to be useful in separating LM from chronically sun-damaged skin [22]. R21 is a monoclonal antibody against adenylyl cyclase, a soluble enzyme that shows good nuclear staining, and has recently been used in the diagnosis of LM [23]. When Mel-5 is used as a rapid immunostain in Mohs surgery, it has been shown to have excellent efficacy and cause less nonmelanocyte collateral staining [24]. This marker, however, is ineffective and is usually associated with high background staining. A rise in giant granules of melanin, macromelanosomes within melanocytes and keratinocytes, has recently been identified as a useful function in distinguishing LM/LMM from solar lentigines [25]. According to Agarwal-Antal et al. [26], invasive melanoma is found in 16% of LM. Diagnostic excisional biopsy with small margins has been considered the gold standard for diagnosing melanoma because incisional biopsy will underestimate the extent of the lesion due to sampling error [27]. A broad shave biopsy extending into the deep papillary dermis or superficial reticular dermis can also be ideal for LM/LMM because it allows for the evaluation of a large piece of tissue [28].

## 4. Noninvasive Procedures

Several noninvasive imaging procedures such as reflectance confocal microscopy (RCM), dermoscopy, and Wood's lamp may improve LM/LMM diagnostic precision, help in biopsy site selection, improve margin delineation, and serve as a useful tool for treatment monitoring [29]. RCM develops horizontal quasi-histological images using near-infrared laser light. RCM increases the accuracy of multiple skin tumor diagnoses [30]. It is very useful for diagnosing and monitoring LM/LMM because it has cellular resolution and allows visualization of very small quantities of melanin that are invisible to dermoscopy or the naked eye [30]. RCM is thus an excellent tool for distinguishing LM/LMM from solar damage and benign macules [31, 32]. RCM is especially useful for identifying amelanotic/hypomelanotic and recurrent LM/LMM lesions located on the head and neck region [33, 34]. RCM enhances the management of challenging lesions by growing the physician's diagnostic belief and diagnostic sensitivity [30]. In fact, for the diagnosis of LM, when compared to dermoscopy (overall sensitivity 0.73; overall specificity 0.84), the RCM is more specific (overall specificity of RCM 0.89) and sensitive (overall sensitivity of RCM 0.93) [35]. Furthermore, combining RCM and dermoscopy improves the accuracy of diagnosis of both of these procedures when used separately for facial tumors [36]. When assessing suspected LM, RCM and histopathology results were found to be consistent in 89 percent of cases by Menge et al. but skin damage may limit the diagnosis' specificity [37]. Dendritic cells, usually large, can be seen on RCM as a result of atypical melanocyte proliferation at the DEJ [32]. Pagetoid distribution of large pleomorphic cells is seen across all layers of the epidermis as LM progresses, causing epidermal disarray. At the dermal-epidermal junction, poorly defined dermal papillae and atypical cells may form bridges that resemble mitochondrial structures [38]. In comparison to nonmelanocytic skin neoplasms, resembling caput medusae, junctional swelling with penetration of the hair follicle was found to be representative of LM/LMM, with an overall specificity of 83% and sensitivity of 96% [39, 40]. RCM may also be used to map the extent of LM/LMM before treatment and to determine margins in ill-defined lesions. The use of videomosaics in conjunction with handheld RCM (HRCM) has allowed for the accurate evaluation of large lesions in curved areas of the body, including the face. HRCM has been shown to be effective in detecting subclinical margins and invasion, making it a useful method for determining the best treatment option [41].

Dermoscopy allows for the visualization of skin structures that are not apparent to the naked eye, enhancing diagnostic precision for both nonpigmented and pigmented lesions. It consists of a polarized or nonpolarized light source attached to a handheld magnifier lens (normally around 10x). Dermoscopy, both nonpolarized and polarized, provides additional information for the LM/LMM diagnosis and has been found to be superior to Wood's lamp inspection in defining the LM/LMM borders [42]. It is important to remember that facial skin has a lot of terminal hair follicles, attenuated rete ridges, and sweat gland ostia when evaluating facial LM/LMM. The presence of a pseudonetwork in facial skin is created by these unusual features: a structureless pigment region disrupted by nonpigmented adnexal openings [43]. Mataca et al. [44] found that the histopathologic diagnostic sensitivity for reflectance confocal microscopy- (RCM-) selected sites were higher than those for dermoscopy-selected sites in a retrospective study.

Wood's light is used for diagnosis and is the therapeutical approach of various tumors, fungal infections, bacterial infections, and pigment diseases. Wood's light can be used to identify subclinical lesions of actinic keratosis after application of 5-aminolevulinic acid (ALA), as it can be used to assess the surgical border in LMM. Although use of Wood's light in LM is not a common approach and even the author had not used it during the case report, literature reviews had many research papers which stated otherwise. The literature states that the surgical borders of LMM can be determined more easily using Wood's light since there is a rise in epidermal melanin in the lesions. Wood's light, on the other hand, was found to detect lesions that could not be seen by the naked eye in just 11.7% of cases in a prospective study [45, 46]. Walsh et al. [46] investigated the precision of preoperative Wood's light test for melanoma in situ margin assessment after excisional biopsy in a prospective study. They concluded that using Wood's light to evaluate subclinical disease in these patients is ineffective due to the high rate of false positives and negatives. This is unsurprising given the presence of multiple activated melanocytes in the vicinity of photodamaged skin. As a result, the use of Wood's lamp to delineate the LM/LMM margins may be reduced, as these melanocytes may be highlighted as well, whether isolated or inside benign lesions. Also, it should be noted that Wood's light does not accentuate dermal melanin, which may lead to false negatives for a deeper atypical melanocytic portion [45].

## 5. Management of LM

### 5.1. Nonsurgical Therapy

Nonsurgical procedures, such as laser treatment, topical imiquimod, cryosurgery, and radiation therapy are also used to treat LM and LMM because of their sensitive anatomic location and common occurrence in the elderly population. There is insufficient evidence to suggest these modalities for widespread use. The inability to histologically analyze the entire specimen is also a concern, given the prevalence of invasive melanoma in 8.1 to 16 percent of tumors initially diagnosed as LM [47, 48]. Another issue when using modalities other than excision is the potential for LM to migrate down the adnexa. A review by Ellis et al. [49] reported about 82% of histologic clearance was recorded involving 264 patients who were treated with different regimens. Since the majority of the reports were case series, with some uncontrolled trials, this study was restricted. Ly et al. [50], on the other hand, conducted an interventional study in which imiquimod 5 percent cream was applied five times weekly for 12 weeks and then excision was performed. About, 53 percent of the patients had achieved histopathologic clearance, with weak correlation between histopathologic and macroscopic clearance. Topical imiquimod has been used as an alternative to surgical procedure both before and after surgery, with mixed results [51, 52]. Topical tazarotene 0.1% gel has also been used in combination with topical imiquimod and alone, resulting in increased inflammation, although this has not been shown to enhance LM clearance efficacy [1]. Ablative lasers such as Er:YAG lasers and carbon dioxide, photodynamic therapy, cryotherapy, electrodesiccation, and curettage and lasers such as Alexandrite lasers and Q-switched Nd:YAG have all been used, but their effectiveness has been inconsistent and there is insufficient evidence to draw meaningful conclusions [53, 54]. Cryosurgery has a recurrence rate of 0–40%, different lasers have a recurrence rate of 0–37.8%, and electrodesiccation and curettage have a recurrence rate of 25–100% [55]. Close monitoring for treatment failure is important when using nonsurgical treatments for LM/LMM, and this can be done clinically using dermoscopy and reflectance confocal microscopy [56, 57].

### 5.2. Surgical Therapy

There are treatment dilemmas involving LM for a variety of reasons. For optimum cosmetic and functional results, the most common position of the head and neck necessitates a tissue saving technique. Compared to nonsurgical treatment options, surgery is the gold standard because it allows for histologic confirmation of full lesion clearance and provides the best evidence for effectiveness with low recurrence rates [55]. However, the lowest recurrence rates of the surgical techniques listed are Mohs micrographic surgery and staged excision with en face or radial sectioning [55]. These methods differ from conventional bread loafing during pathologic sectioning, in which only less than 1% of the peripheral margin is histologically investigated, and standard excision with fixed margins. Mohs micrographic surgery involves the surgical removal of tangential disclike samples under local anesthesia, which are then handled with en face parts to allow for 100% surgical margin inspection. This procedure has the benefits of tissue preservation by removing just a small amount of normal tissue around the lesion, as well as increased effectiveness and lower treatment costs by removing the lesion and repairing it on the same day. During Mohs surgery on frozen parts, rapid immunostains, most commonly Melan A/MART 1, are commonly used to enhance detection of irregular melanocytes [20].

## 6. Future Perspective

Machine learning (ML) is an artificial intelligence technique that uses computer algorithms to assist clinicians in making clinical decisions. Deep learning is a fascinating subfield of machine learning in which massive databases can be scaled, allowing them to advance with more data [58]. Deep learning convolutional neural networks (CNNs) have improved ML's melanoma screening performance even further, outperforming some dermatologists [59]. Although certain shortcomings have to be resolved, these algorithms can enhance LM/LMM diagnosis in the future [60]. A CNN was used by Winkler et al. [61] to diagnose different melanoma subtypes, including LMM. A dermatoscopic image package with 30 LMM and 100 benign lesions was used by the researchers, such as nevi, seborrheic keratosis, and macular solar lentigines, which could be matched for position and morphology. Although the authors accept that their dermatoscopic images were of higher quality than those obtained in a clinical routine environment, the results are promising. Furthermore, the majority of the photos were taken from patients with light skin. Since images of LMM in people of other ethnic backgrounds are rare, this may imply additional drawbacks for CNN pattern recognition. Furthermore, some features of pigmented skin lesions prevent ML examination [62, 63]. The most important is the difficulty in determining the lesion's boundary (hair and lesions appearing in volar skin, lack of surrounding normal skin, and lack of pigmentation). Another major drawback is the appearance of large lesions that do not fit into the field of view of the dermatoscopic camera. Furthermore, a study by Gonzalez-Cruz et al. [64] considered the limitations of image collection for ML research. As a result, while ML and CNN are likely to play an essential role in the potential management of LM/LMM, there are still limitations that must be overcome by the use of broader image datasets that best reflect various skin forms, such as benign lesions and photographs taken in an unregulated manner with consumer cameras.

## 7. Conclusion

As the prevalence of LM and LMM rises around the world, dermatologists must maintain a high index of suspicion in order to make an early diagnosis of this often-difficult condition. Reflectance confocal microscopy and dermoscopy are valuable adjuncts for better diagnosis when paired with recently described features. Melanocyte immunohistochemistry and newer markers such as anti-adenylyl cyclase antibodies can help distinguish LM/LMM from background actinic harm. Surgical care for LM and LMM remains the gold standard, with recently defined margin management procedures including Mohs micrographic surgery and staged excision with radial sectioning of margins showing the lowest recurrence rates. Nonsurgical therapies such as laser therapy, radiation therapy, and imiquimod cream have the potential to be used as a primary or adjunctive treatment, but further evidence of effectiveness is required.

## Data Availability

All data are included within the article.
